# Estimating breast tissue-specific DNA methylation age using next-generation sequencing data

**DOI:** 10.1186/s13148-020-00834-4

**Published:** 2020-03-12

**Authors:** James R. Castle, Nan Lin, Jinpeng Liu, Anna Maria V. Storniolo, Aditi Shendre, Lifang Hou, Steve Horvath, Yunlong Liu, Chi Wang, Chunyan He

**Affiliations:** 1 University of Kentucky Markey Cancer Center, 800 Rose Street, Lexington, KY 40536 USA; 2 Susan G. Komen Tissue Bank at Indiana University Simon Cancer Center, Indianapolis, IN USA; 3 Department of Biomedical Informatics, The Ohio State University, Columbus, OH USA; 4 Center for Population Epigenetics, Robert H. Lurie Comprehensive Cancer Center and Department of Preventive Medicine, Northwestern University Feinberg School of Medicine, Chicago, IL USA; 5 Department of Biostatistics, UCLA School of Public Health, Los Angeles, CA USA; 6 Department of Molecular and Medical Genetics, Indiana University School of Medicine, Indianapolis, IN USA; 7 Department of Internal Medicine, Division of Medical Oncology, University of Kentucky College of Medicine, Lexington, KY USA

**Keywords:** DNA methylation, Aging, Epigenetic age, Breast, Next-generation sequencing

## Abstract

**Background:**

DNA methylation (DNAm) age has been widely accepted as an epigenetic biomarker for biological aging. Emerging evidence suggests that DNAm age can be tissue-specific and female breast tissue ages faster than other parts of the body. The Horvath clock, which estimates DNAm age across multiple tissues, has been shown to be poorly calibrated in breast issue. We aim to develop a model to estimate breast tissue-specific DNAm age.

**Methods:**

Genome-wide DNA methylation sequencing data were generated for 459 normal, 107 tumor, and 45 paired adjacent-normal breast tissue samples. We determined a novel set of 286 breast tissue-specific clock CpGs using penalized linear regression and developed a model to estimate breast tissue-specific DNAm age. The model was applied to estimate breast tissue-specific DNAm age in different breast tissue types and in tumors with distinct clinical characteristics to investigate cancer-related aging effects.

**Results:**

Our estimated breast tissue-specific DNAm age was highly correlated with chronological age (*r* = 0.88; *p* = 2.9 × 10^−31^) in normal breast tissue. Breast tumor tissue samples exhibited a positive epigenetic age acceleration, where DNAm age was on average 7 years older than respective chronological age (*p* = 1.8 × 10^−8^). In age-matched analyses, tumor breast tissue appeared 12 and 13 years older in DNAm age than adjacent-normal and normal breast tissue (*p* = 4.0 × 10^−6^ and 1.0 × 10^−6^, respectively). Both HER2+ and hormone-receptor positive subtypes demonstrated significant acceleration in DNAm ages (*p* = 0.04 and 3.8 × 10^−6^, respectively), while no apparent DNAm age acceleration was observed for triple-negative breast tumors. We observed a non-linear pattern of epigenetic age acceleration with breast tumor grade. In addition, early-staged tumors showed a positive epigenetic age acceleration (*p* = 0.003) while late-staged tumors exhibited a non-significant negative epigenetic age acceleration (*p* = 0.10).

**Conclusions:**

The intended applications for this model are wide-spread and have been shown to provide biologically meaningful results for cancer-related aging effects in breast tumor tissue. Future studies are warranted to explore whether breast tissue-specific epigenetic age acceleration is predictive of breast cancer development, treatment response, and survival as well as the clinical utility of whether this model can be extended to blood samples.

## Introduction

DNA methylation (DNAm) levels in specific sets of cytosine-phosphate-guanines (CpGs) in the human genome can be used to establish an epigenetic biomarker of biological age [1–6], known as an “epigenetic clock,” where the resulting age estimate is commonly referred to as “epigenetic age” or “DNAm age.” Increasing evidence suggests that many facets of aging are epigenetic [7, 8] and that DNAm age captures both the genetic and environmental influences across time on cellular functions [1, 2, 4, 6]. Furthermore, the difference between DNAm and chronological ages, known as epigenetic age acceleration, has been shown to be associated with various health conditions and outcomes, including obesity [9], lifetime stress [10], HIV infection [11, 12], cognitive impairment [13–15], cancer [16, 17], and mortality [18–20].

A multi-tissue DNAm age estimator was developed recently using a large dataset of DNAm profiles measured on the Illumina Methylation 27K and 450K microarray platforms (Illumina Inc., San Diego, CA, USA). This model, known as the Horvath clock model, displays a remarkable accuracy in predicting chronological age across multiple tissue types using the methylation levels of only 353 CpG loci in the human genome [6]; however, DNAm ages estimated with this model were not well calibrated in several tissue types, including breast tissue, uterine endometrium, dermal fibroblasts, skeletal muscle tissue, and heart tissue [6]. Further studies using the Horvath clock model suggested that female breast tissue ages faster than other parts of the body [6, 21, 22]. As age is an established risk factor for breast cancer, a breast tissue-specific DNAm age estimator may be more appropriate in studying aging effects on breast tissue and their contribution to breast cancer development; however, no model has been developed to estimate breast tissue-specific DNAm age. This study aims to develop a model that estimates breast tissue-specific DNAm age. We hypothesize that a novel set of breast-tissue specific CpG markers can be identified to estimate DNAm age accurately in breast tissue.

The increasing availability of next-generation sequencing data also calls for method development that uses DNA methylation sequencing data in targeted tissue to estimate tissue-specific DNAm age. In this study, we utilized a large data set of breast tissue-specific DNAm profiles generated using the Illumina TruSeq Methyl-Capture EPIC Library Prep Kit and next-generation sequencing technology (EPIC-seq), comprising 459 normal (K) breast tissue samples from healthy women, and 107 tumor (T) and 45 matched adjacent-normal (N) tissue samples from breast cancer patients. We used a random subset of normal breast tissue samples (*N* = 368) to construct a breast tissue-specific model of DNAm age estimation. We performed a pathway analysis of the genes annotated to the CpGs identified in the model to assess biological influences of aging in healthy normal breast tissue. Further, we applied the model to the remaining data sets of normal (*N* = 91), tumor, and adjacent-normal breast tissue. The resulting DNAm age estimates were used to investigate epigenetic age acceleration in different breast tissue types as well as in breast tumors with distinct clinical characteristics.

## Results

### Breast tissue-specific model of epigenetic age

**Fig. 1:**
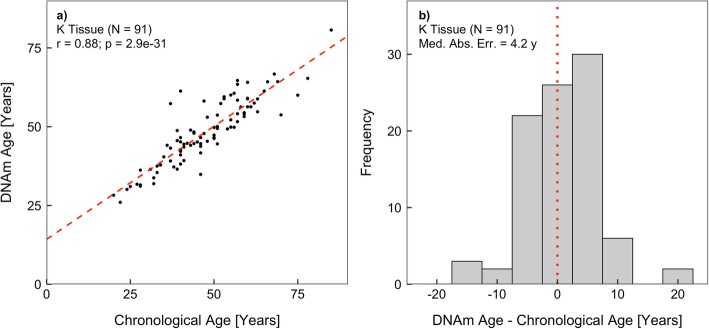
The performance of breast tissue-specific DNAm age model in the normal breast tissue testing data set: **a** correlation between DNAm age and chronological age and **b** distribution of model estimation error, defined as the difference between DNAm and chronological age

The elastic net regression algorithm generates sparsity from its regularization terms and, as a consequence, performs automatic feature selection [23]. Of the 2,471,574 candidate CpGs entering the algorithm, 286 were selected as breast tissue-specific clock CpGs. A model was constructed for the estimation of breast tissue-specific DNAm age using these identified clock CpGs and regression coefficients (Additional file 1). We evaluated the accuracy of the model using two measures: the Pearson correlation coefficient between DNAm age and chronological age and the absolute median predictive “error” defined as the median absolute difference between the estimated DNAm age and chronological age. While the accuracy in training data set is likely overly optimistic due to overfitting (*r* = 0.99; *p* < 1 × 10^−32^; median absolute error = 1.1 years), the assessments in the testing data set are unbiased. In the testing data set, breast tissue-specific epigenetic age was found to be highly correlated with chronological age (*r* = 0.88; *p* = 2.9 × 10^−31^, Fig. 1). The absolute median predictive error in the testing data set was 4.2 years, indicating that DNAm age differed from chronological age by less than 4.2 years in 50% of the subjects.

**Table 1:** Age distributions of breast tissue data sets in the study

Tissue type	*N*	Mean age (years)	SD (years)
Normal	459	47.7	13.4
Training set	368	47.7	13.5
Testing set	91	47.7	12.9
Adjacent normal*	45	53.1	12.1
Tumor*	107	55.3	13.6
HER2+	21	56.8	16.9
ER+ or PR+/HER2-	42	54.4	11.7
ER-/PR-/HER2-	15	53.9	12.4

*HER2* epidermal growth factor receptor 2, *ER* estrogen receptor, *PR* progesterone receptor

*Age distribution is significantly different from the normal breast tissue testing data set (*p* < 0.05)

### Breast tissue-specific clock CpGs

The set of 286 breast tissue-specific clock CpGs comprises 190 and 96 CpGs whose DNAm levels are positively and negatively associated with chronological age, respectively. Positively associated CpGs were primarily located in CpG islands (84% in islands, 7% in shores, and 9% in open sea), while negatively associated CpGs were more interspersed (66% in islands, 15% in shores, and 19% in open sea). An Ingenuity Pathway Analysis (IPA) [24] of the genes that co-locate with the 286 clock CpGs showed significant enrichment in functions including gene expression, cellular development, and cell morphology. While positively associated CpGs suggested top canonical pathways including epidermal growth factor (EGF) signaling and estrogen-receptor (ER) signaling, negatively associated CpGs suggested canonical pathways including Ataxia-Telangiectasia Mutated (ATM) signaling and apoptosis signaling.

**Fig. 2:**
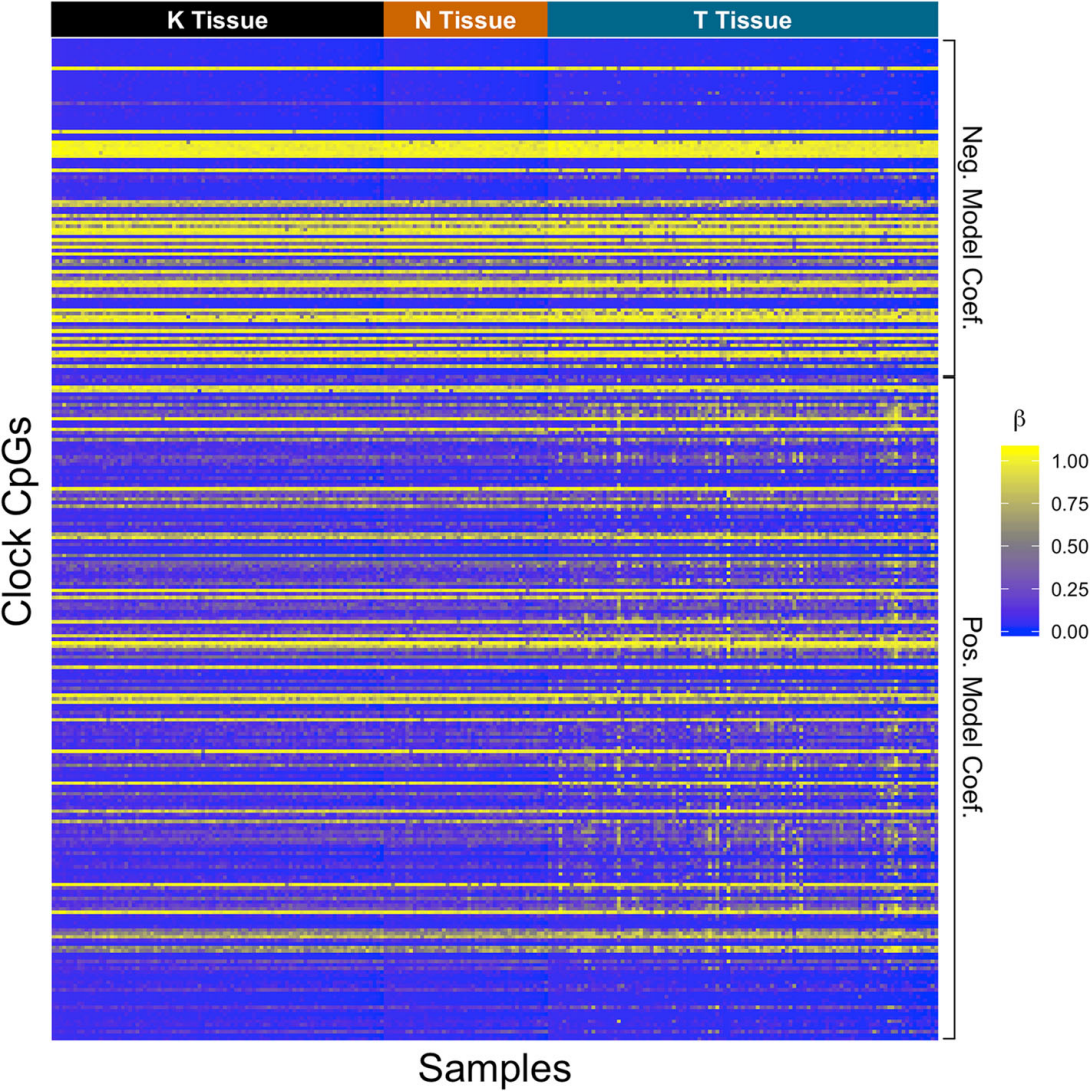
Heatmap of *β* values of the clock CpGs across all samples. Samples are arranged by age within each tissue type in the columns from youngest to oldest (left to right) and clock CpGs are arranged by model coefficient value in the rows from smallest to largest (top to bottom)

**Fig. 3:**
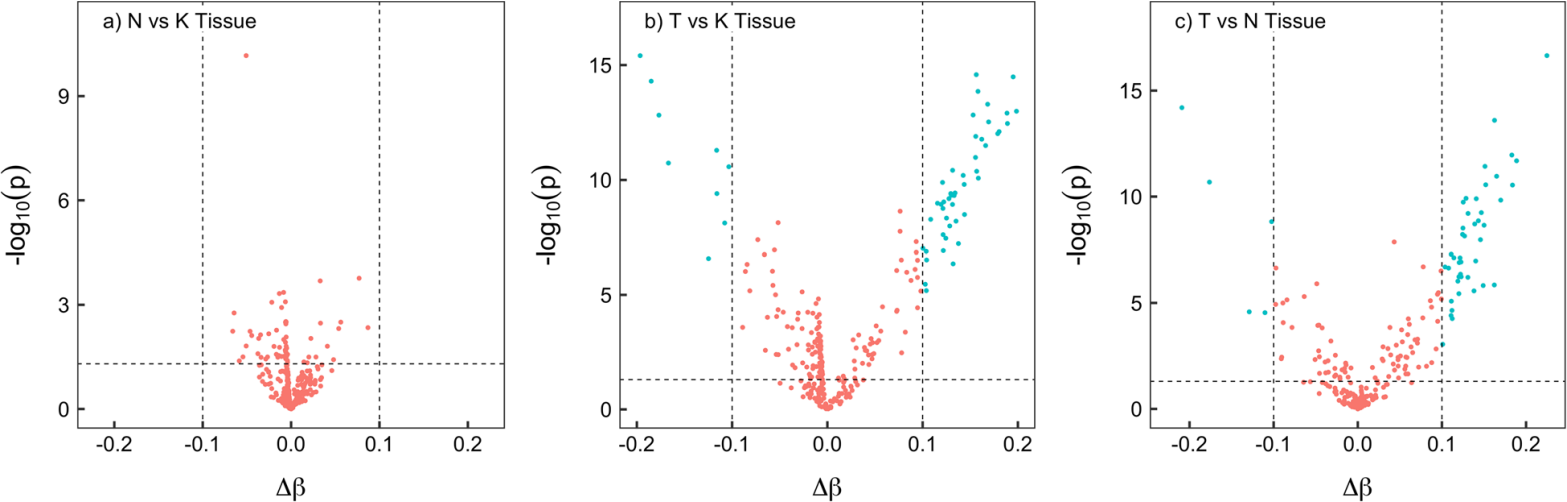
Volcano plots of differentially methylated clock CpGs in pairwise comparisons **a** adjacent-normal (N) vs normal (K); **b** tumor (T) vs normal (K); and **c** tumor (T) vs adjacent-normal (N) breast tissue. Differentially methylated clock CpGs (in cyan) are identified if |∆β| > 0.1 and *t* test *p* < 0.05

Age-adjusted DNAm levels of the 286 clock CpGs across all samples from three breast tissue types are illustrated in a heat map (Fig. 2). A clear distinction can be seen in the DNAm levels between breast tumor tissue and normal or adjacent normal tissue, where most of the positively associated CpGs (with age) were hypermethylated in breast tumor tissue when compared to normal and adjacent-normal tissue. This pattern was better quantified when differentially methylated clock CpGs were identified in pairwise tissue comparisons (Fig. 3). In the comparison between tumor and normal breast tissue, 56 clock CpGs were differentially methylated, of which 9 were hypomethylated and 47 were hypermethylated in tumor breast tissue. Similarly, in the comparison between tumor and adjacent-normal breast tissue, 48 clock CpGs were differentially methylated, of which 5 were hypomethylated and 43 were hypermethylated in tumor breast tissue. There was a 94% overlap of CpGs between the two comparisons. An Ingenuity Pathway Analysis of the genes annotated to these differentially methylated CpGs suggested nicotinamide adenine dinucleotide (NAD) biosynthesis pathway for the hypermethylated clock-CpG genes in tumors, while no statistically significant pathways were identified for the hypomethylated clock-CpG genes. No statistically significant and differentially methylated CpGs were identified in the comparison between normal and adjacent-normal breast tissue.

### DNAm age estimation in different breast tissue types

**Fig. 4:**
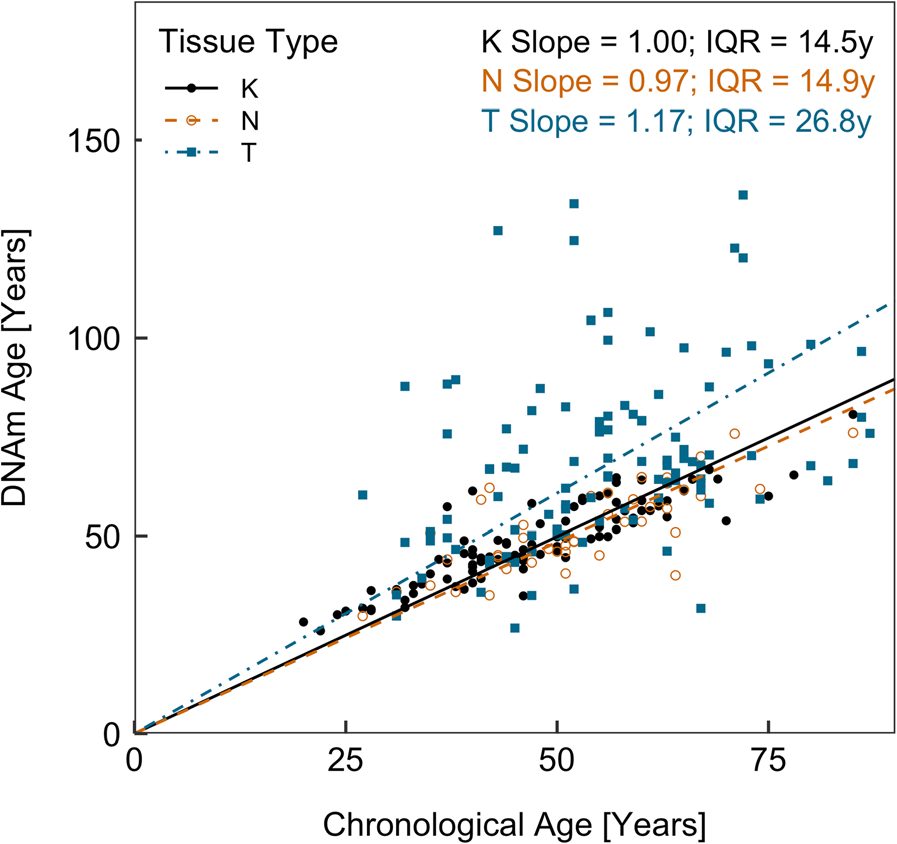
Scatter plot of DNAm age and chronological age in three tissue types. A regression line is fitted for each tissue type. The slope of each regression line indicates the change in DNAm age corresponding to each unit change in chronological age, or epigenetic age acceleration rate. Interquartile ranges (IQR) are reported for each tissue type

Using our model, we estimated DNAm age in tumor and adjacent-normal breast tissue samples and further calculated respective epigenetic age acceleration difference (EAAD) values. In general, breast tumor tissue appeared to have much larger variation in both DNAm age and EAAD values compared to normal and adjacent-normal tissue (Figs. 4 and 6a). We fitted a fixed-intercept linear model for each tissue type by regressing DNAm age on chronological age (Fig. 4), where the slope of each line indicates the change in DNAm age corresponding to each unit change in chronological age and provides a measure of epigenetic age acceleration rate. We found that tumor breast tissue exhibited a higher rate (slope = 1.17) than both normal and adjacent-normal tissue (slope = 1.00 and 0.97, respectively). While the slope differences are not statistically significant, it suggests that breast tumor tissue might be aging at a faster rate than normal and adjacent-normal tissue (*p* = 0.47 and 0.67, respectively). We further assessed the magnitude and direction of epigenetic age acceleration in each tissue type using EAAD values (Fig. 6a). We found that there was no statistically significant epigenetic age acceleration in either breast normal tissue (median EAAD = 0.7 years, *p* = 0.18) or adjacent-normal tissue (median EAAD = − 2.3 years, *p* = 0.07). However, breast tumor tissue had a pronounced epigenetic age acceleration towards older ages (median EAAD = 6.8 years, *p* = 1.8 × 10^−8^).

**Fig. 5:**
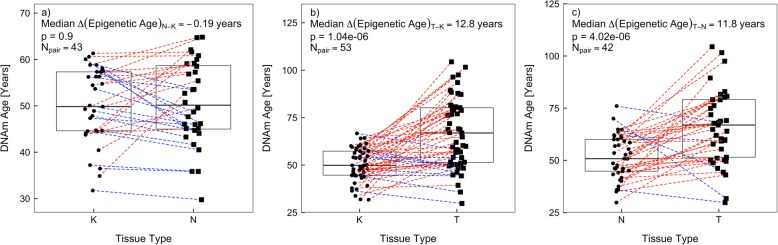
Pairwise analysis of DNAm age between different breast tissue types matched on chronological age: **a**, adjacent-normal (N) vs normal (K); **b**, tumor (T) vs normal (K); and **c**, tumor (T) vs adjacent-normal (N) breast tissue. In each plot, dotted lines joining points in each tissue group denote paired samples. Boxes in each plot represent the first, second, and third quartiles of each distribution. Wilcoxon signed-rank tests were used to obtain *p* values

**Fig. 6:**
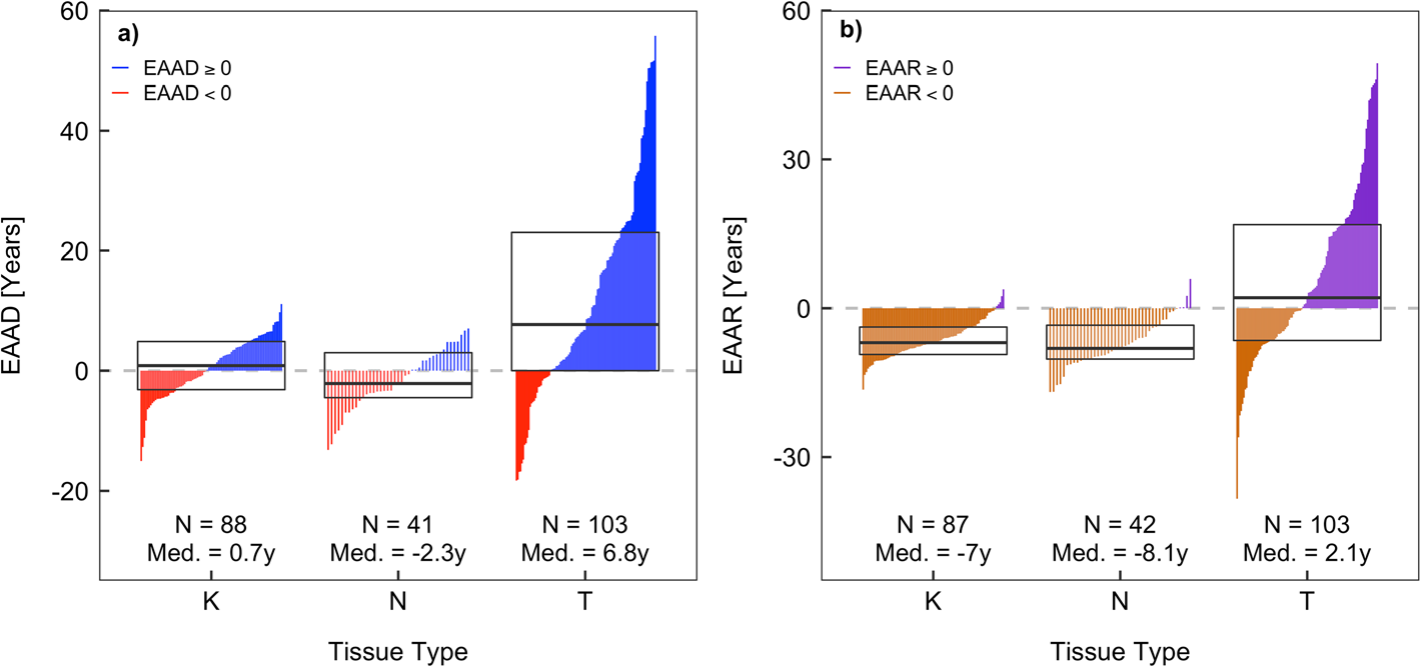
Distributions of breast tissue-specific epigenetic age acceleration for normal, adjacent-normal, and tumor breast tissue: **a** epigenetic age acceleration differences (EAAD) and **b** epigenetic age acceleration residuals (EAAR). Boxes in each plot represent the first, second, and third quartiles of each distribution. Vertical lines within each box represent individual sample epigenetic age acceleration values and are colored based on their sign. Red (orange) lines represent negative EAAD (EAAR) values, while blue (purple) lines represent positive EAAD (EAAR) values

As our breast tumor and adjacent-normal tissue samples are significantly older than our normal tissue samples, confounding by chronological age needs to be considered when comparing DNAm age and its related measures across different tissue types. We utilized two approaches to control for confounding by chronological age when comparing tissue types. First, we performed chronological age-matched analyses between tissue types (Fig. 5). We found that breast tumor tissue had much older DNAm age than age-matched normal and adjacent-normal tissue (median ∆ (DNAm age) = 12.8 and 11.8 years, *p* = 1.0 × 10^−6^ and 4.0 × 10^−6^, respectively), while there was no significant difference in DNAm age between age-matched normal and adjacent-normal tissue (median ∆ (DNAm age) = − 0.2 years, *p* = 0.90). Secondly, we compared epigenetic age acceleration residual (EAAR) values across different tissue types (Fig. 6b). We observed that breast tumor tissue had higher EAAR values than normal and adjacent-normal tissue (median EAAR = 2.1, − 7.0, and − 8.1 years, respectively), indicating tumor tissue was relatively 9.1 and 10.2 years epigenetically older than normal and adjacent-normal tissue (*p* = 3.0 × 10^−8^ and 1.6 × 10^−7^, respectively). These results were consistent with those from chronological age-matched analyses, suggesting tumor breast tissue exhibits significant epigenetic age acceleration towards older ages when compared to normal and adjacent-normal breast tissue.

### DNAm age estimation in breast tumors with distinct clinical features

**Fig. 7:**
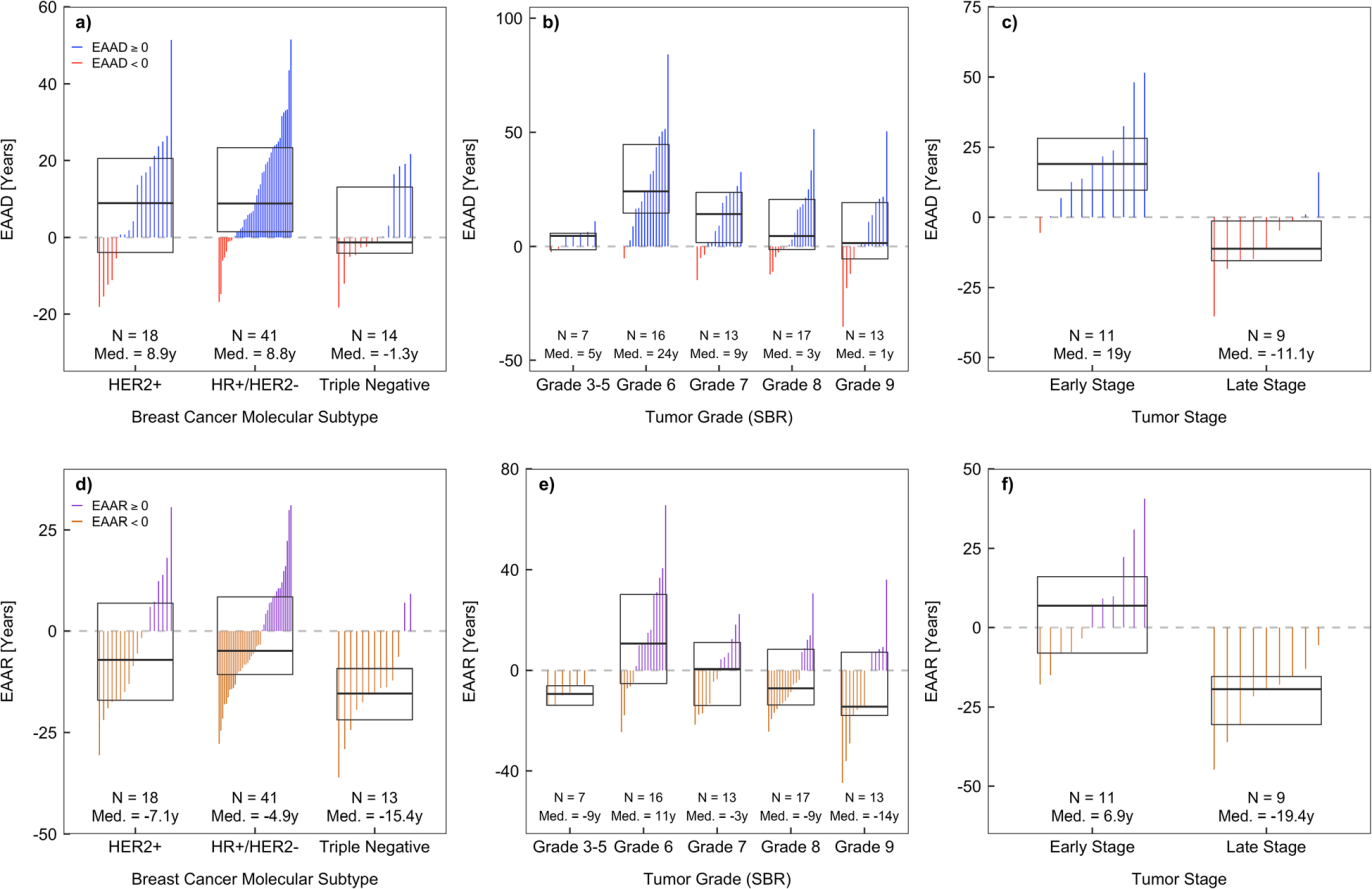
Distributions of breast tissue-specific epigenetic age acceleration in each tumor group with distinct clinical features. **a**–**c** epigenetic age acceleration differences (EAAD) and **d**–**f** epigenetic age acceleration residuals (EAAR). Boxes in each plot represent the median and interquartile range for each distribution. Vertical lines within each box represent individual sample epigenetic age acceleration values and are colored based on their sign. Red (orange) lines represent negative EAAD (EAAR) values, while blue (purple) lines represent positive EAAD (EAAR) values

We further explored DNAm age in breast tumor subgroups with distinct clinical features, including molecular subtype, tumor grade, and tumor stage. We defined three molecular tumor subtypes based on HER2 and hormone receptor (HR) status: HER2+, HR+ (ER+ or PR+)/HER2-, and HER2-/ER-/PR- (triple negative, TNBC). We found that both HER2+ and HR+/HER2- tumor subtypes had significant epigenetic age accelerations towards older ages (median EAAD = 8.9 and 8.8 years, *p* = 0.04 and 3.8 × 10^−6^, respectively), while TNBC showed no significant epigenetic age acceleration (median EAAD = − 1.3 years, *p* = 0.86) (Fig. 7a). A non-linear pattern was observed between epigenetic age acceleration and tumor Scarff-Bloom-Richardson (SBR) grade (Fig. 7b). Epigenetic age acceleration was near zero for grades 3–5, peaked around grade 6, and then decreased as grade increased. A significant epigenetic age acceleration towards older ages was observed for grades 6 and 7 (median EAAD = 24.0 and 9.1 years, *p* = 1.5 × 10^−4^ and 0.03, respectively). Early-stage breast tumors (stage II) showed a significant epigenetic age acceleration towards older ages (median EAAD = 19.0 years, *p* = 0.003), while late-stage breast tumors (stages III and IV) had non-significant epigenetic age acceleration towards younger ages (median EAAD = − 11.1 years, *p* = 0.10) (Fig. 7c).

We compared epigenetic age acceleration across different tumor subgroups, controlling for confounding by chronological age using EAAR values. We observed that HER2+ and HR+/HER2- samples were relatively 8.2 and 10.5 years older than TNBC samples (*p* = 0.11 and 0.02, respectively) (Fig. 7d). In addition, age-adjusted epigenetic age acceleration showed a similar relationship with tumor grade, where the largest acceleration was observed around grade 6, which then decreases as grade increases (Fig. 7e). Early- and late-stage breast tumors appeared to have opposite directions in epigenetic age acceleration and early-stage tumors were relatively 26.3 years older than late-stage tumors (*p* = 0.001).

### Comparison to the Horvath clock model

**Fig. 8:**
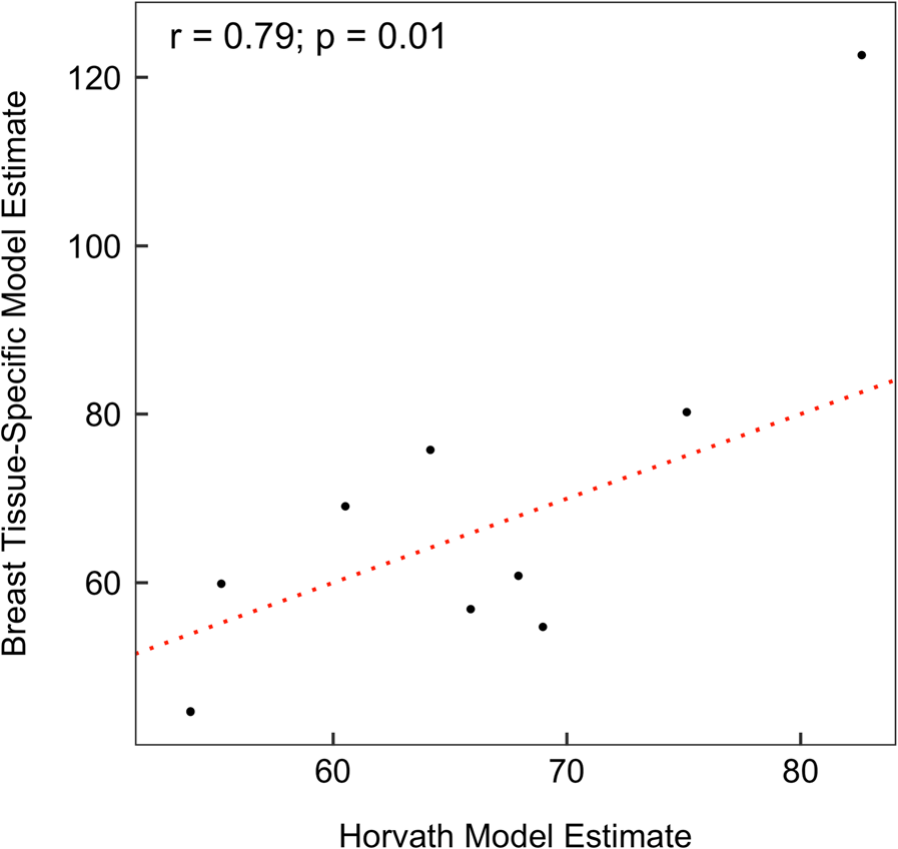
Concordance of DNAm ages estimated by our breast tissue-specific model and by the Horvath clock model. Horvath clock model estimates were obtained using data profiled with the Infinium MethylationEPIC BeadChip technology, while our model estimates were obtained using data profiled with the TruSeq Methyl-Capture EPIC sequencing technology in same samples. The dotted line indicates complete concordance

Our model was trained using data from targeted normal breast tissue, while the Horvath clock model was developed using data across multiple tissues. The two models likely are not comparable because of the difference in reference tissues. We found only one clock CpG directly overlaps between our model and the Horvath clock model. Despite the expansive coverage of the EPIC-seq platform, only 268 of the 353 Horvath clock CpGs were profiled in our sequencing dataset. This limits our ability to directly compare DNAm age estimates from the Horvath clock model and our breast-tissue specific model using the same sequencing data. Previous studies have shown that the Horvath clock model can be reliably applied to EPIC-Array platform data with minimal induced variance [25, 26]. We thus compared DNAm age estimates from our breast tissue-specific model to those from the Horvath clock model in a small subset of samples (*N* = 9) for which we obtained DNAm profiles using both the EPIC-Array and EPIC-Seq technologies. Although DNAm age estimates from the two models were in good concordance (*r* = 0.79, *p* = 0.01), we observed that the median absolute difference in DNAm age was 9.0 years between the two models (Fig. 8).

## Discussion

DNAm age is an epigenetic marker of biological aging that reflects age-related cumulative changes in DNA methylation influenced by both environmental and genetic risk factors. The Horvath method has been widely used to estimate DNAm age based on methylation levels of 353 clock CpGs measured from earlier array technologies [6]. Compared to recent data from sequencing technologies, these array data have a limited coverage and resolution on genome-wide DNA methylation. Thus, the increased availability of sequencing data may provide an unprecedented opportunity to refine the Horvath clock model by considering more CpGs in training and selecting optimal clock CpGs for DNAm age estimation. On the other hand, the Horvath clock model is poorly calibrated in breast tissue with a high error rate and breast tissue appearing older than other parts of the body [6]. Therefore, it is reasonable to single out on breast tissue and develop a breast tissue-specific model to more accurately estimate DNAm age in breast tissue. To the best of our knowledge, this is the first study that develops a breast tissue-specific model for DNAm age estimation using DNAm sequencing data. We have shown that the tissue-specific model developed in the study not only has a higher accuracy in DNAm age estimation for normal breast tissue, but also is applicable to other breast tissue types and yields biologically meaningful results.

Our breast tissue-specific model selected 286 clock CpGs for DNAm age estimation, the number of which is about 20% less than the 353 clock CpGs in the Horvath clock model. Despite the smaller number of clock CpGs, our model has a significantly improved predictive accuracy in breast tissue (*r* = 0.88, median error = 4.2 years), compared to the Horvath clock model (*r* = 0.73, median error = 8.9 years). This drastic improvement in accuracy is likely a result of the optimal selection of clock CpGs in our model that better capture tissue-specific changes. The Horvath clock model is likely less sensitive to tissue-specific changes, given that its reference training data is across multiple tissue types. This could lead to an “averaging out” of tissue-specific changes in order to achieve a pan-tissue epigenetic clock. Further, the Horvath clock model is not well calibrated in female breast tissue, which will add additional noise to studies involving this tissue type. Since our model targets breast tissue specifically, it reduces both the averaging-out and induced noise limitations of the Horvath clock model in breast tissue. To ensure our model selected the most relevant set of CpGs, we conducted a sensitivity analysis where the model was retrained on two sets of CpGs that underwent “loose” and “strict” levels of quality control (QC). For the strict-QC model, CpGs were restricted to have <1% missingness across samples to reduce the dependence on imputation. A total of 1.2 million CpGs remained after QC. Model training selected 247 clock CpGs, of which 122 overlapped with the current model. The strict model had a slight reduction in predictive accuracy (*r* = 0.87, median error = 4.5 years). For the loose-QC model, CpGs had no restrictions on missingness, and all missing values were simply imputed. A total of 3.3 million CpG probes remained after QC. Model training selected 280 clock CpGs, of which 191 overlap with the current model. This loose model had a slight increase in predictive accuracy (*r* = 0.89, median error = 3.5 years), which might be attributed to overfitting as a result of a large increase in the number of imputed CpGs. We applied both the strict-QC and loose-QC models to all other analyses in the study and found essentially identical results. The consistency of results between the three model instances supports the robustness of the model developed in our study.

Although only one clock CpG directly overlaps between the 286 and the 353 clock CpGs in our model and the Horvath clock model, the DNAm age estimates from both models were still in good concordance (*r* = 0.79), suggesting both sets of clock CpGs might capture biological pathway or functions related to general aging processes. Indeed, the Ingenuity Pathway Analysis of annotated genes in both models suggests enrichment of functions including cell death and survival, cellular growth and proliferation, tissue development, and cancer; however, the annotated genes in our model may be more related to tissue-specific aging through multiple stages of cell life. Of particular interest, the top clock CpGs positively associated with age in our model and their annotated genes are enriched in EGF and estrogen-receptor signaling. It is hypothesized that hormone cycling during menstruating years is responsible for the observed accelerated aging in female breast tissue with respect to the rest of the body [6, 21, 22]. Further, EGF overexpression is observed in all subtypes of breast cancer and has been shown to be associated with larger tumor size, poor differentiation, and poor outcomes [27–29]. Top clock CpGs negatively associated with age and their annotated genes are enriched in ATM and apoptosis signaling, both of which are linked to genome integrity and aging [30] as well as sustaining breast-cancer tumorigenicity [31] and tumor morphology [32]. The correlation of these cancer-related CpGs with DNAm age provides a mechanistic link through which aging contributes to cancer development.

The DNA methylation patterns of our 286 clock CpGs across all samples and tissue types showed a clear separation of tumor breast tissue from normal or adjacent-normal breast tissue. Differentially methylated clock CpGs between tumor and normal or adjacent-normal breast tissue are enriched in cancer-related pathways. Hypermethylated clock CpGs in tumors were related to NAD biosynthesis, which declines during the aging process [33]. It has been proposed that this decline triggers the interaction between DCB1 and PARP1, which decreases the frequency of DNA damage repair [33]. These hypermethylated clock CpGs suggest potential biological functions involved in acceleration of the aging process in breast tumor tissue relative to normal and adjacent-normal breast tissue, which is consistent with our other observations that breast tumor tissue has a positive acceleration of DNAm age.

When considering all 286 clock CpGs jointly to estimate DNAm age in our model, we found the DNAm age could easily distinguish breast tumor tissue from normal and adjacent-normal tissue. As chronological age was increased, DNAm age in breast tumor tissue increased at a much higher rate (by ≈ 17%) compared to almost no increase in normal and adjacent-normal breast tissue. On average, breast tumor tissue was about 7 years older in DNAm age than its chronological age, while no significant age acceleration was observed for normal and adjacent-normal breast tissue. The larger inter-sample variation of DNAm age estimates in breast tumor tissue might be consistent with well-known tumor heterogeneity and its underlying complex etiology. In addition, breast tumor tissue was approximately 13 and 12 years older than normal and adjacent-normal breast tissue in DNAm age, respectively, in age-match analysis, also indicating a much higher age acceleration in tumor tissue. These results suggest that DNAm age is not only a marker for aging, but also a promising marker for breast cancer development.

Since our model is not directly comparable to the Horvath model due to differences in the scope of our training data sets (breast tissue-specific vs pan tissue), it raises questions as to whether our model captures aging mechanisms that are unique to or behave differently in breast tissue when compared to those from the Horvath clock model. Indeed, pathway analyses revealed that our model includes some clock CpGs and annotated genes that may be specific to the breast tissue aging process. Furthermore, our model yields a smaller error rate of DNAm age estimation in breast tissue compared to the Horvath clock model. The more accurate estimation of DNAm age would lead to more study power to access epigenetic age acceleration in a given tissue or to compare relative epigenetic age acceleration across different tissue types. Thus, our model is likely to be more relevant and accurate when assessing aging effects on the development of breast diseases such as breast cancer. Indeed, our study has sufficient power to detect moderate differences in DNAm age acceleration between tissue types. Specifically, our study has 80% power to detect a difference in DNAm age acceleration of 1.1, 1.3, and 1.7 years between tumor and normal, between adjacent-normal and normal, and between tumor and adjacent-normal breast tissue, respectively.

Breast cancer is a heterogeneous disease characterized by distinct clinical and pathological features and molecular subtypes [34], reflecting different underlying molecular mechanisms for cancer development. Thus, it is of interest to further explore the performance of DNAm age estimation in these breast tumor subgroups. We found that both HER2+ and HR+/HER2- tumors, which are more responsive to hormone recycling, showed a similar magnitude of positive age acceleration (approximately 10 years). This is in line with the results of our earlier pathway analysis showing enrichment in the EGF and estrogen-receptor signaling pathway for our selected clock CpGs. In contrast, we observed a negligible age acceleration in TNBC, which is an aggressive subtype of the disease with poor outcomes [35]. Recent studies suggest that cancer stem cells are enriched in TNBC and play an important role in tumorigenesis and tumor biology of this subtype [36–38]. Cancer stem cells have the unique ability for both self-renewal as well as the ability to re-establish a heterogeneous population of tumor cells (potential to differentiate). Similar to embryonic stem cells, which have been shown to have a DNAm age close to zero [6], we expect that the DNAm age for cancer stem cells to be very young. This, in turn, may “cancel out” the age acceleration in the developed tumor cells in TNBC samples and explain the lack of age acceleration observed. Previous studies using the Horvath clock model found consistent relative epigenetic age acceleration differences for HR+/HER2- and TNBC tumors [6, 39], supporting the validity of our results in this study.

Further inspection of DNAm age with tumor morphology (grade and stage) provided additional insights on the relation of epigenetic age acceleration and the ability of tumor cells to proliferate, differentiate, and potentially metastasize. We observed a non-linear epigenetic age acceleration relationship with Scarff-Bloom-Richardson grade, where epigenetic age acceleration is near zero for grades 3–5, then peaks near grade 6, and then gradually decreases for higher grades. Compared to low-grade tumors, high-grade tumors are undifferentiated, or poorly differentiated, and are more likely to grow and proliferate. Thus, it is conceivable that epigenetic age acceleration in high-grade tumors tends towards younger or smaller values compared to low-grade tumors after certain stage. These speculations are further supported by our epigenetic age acceleration results in relation to tumor stage, where we observed a large, positive age acceleration in early-stage tumors but a negative age acceleration in late-stage/advanced tumors. Similar to high-grade tumors, advanced and late stage tumors are more likely to be poorly differentiated and have a greater potential to metastasize to distal locations. A previous study using the Horvath clock model suggested a similar negative association between stage and age acceleration in thyroid cancer [6]. These observations are in line with the theory that DNAm age is in some way related to the biological processes underlying development, cell differentiation, and the maintenance of cellular identity. Therefore, epigenetic age acceleration may capture both intracellular changes in losing cellular identity and changes in cell composition [40]. While providing interesting insights, these findings were obtained using a relatively small number of case samples and need to be validated in larger studies.

Our study has several strengths. First, we measured genome-wide DNA methylation using next-generation sequencing technology, which provides a much higher coverage and resolution of the human genome compared to previous data from array technology. This allows for more CpGs to be considered when building our model, which ultimately leads towards choosing the optimal set of CpGs that accurately captures age-related DNAm changes. Second, our data is from targeted breast tissue and therefore allows us to capture age-related DNAm changes specific to female breast tissue. The tissue specificity of our model overcomes limitations (e.g., induced noise) set by the poor calibration of breast tissue in the Horvath clock model and allows for more powerful studies. Third, we are able to apply the developed model to different breast tissue types including breast normal, adjacent-normal, and tumor tissue and demonstrate that DNAm age changes across tissue type. Lastly, with our model and data, we are able to examine the relationship between DNAm age and several tumor clinical features, providing insights on epigenetic aging during tumor progression. We also acknowledge the limitations of our study, including small sample size in the tumor subgroup analyses and the limited adjacent-normal breast tissue samples. Larger and independent samples are needed to validate the findings of this study. In addition, since we include only breast tissue in this study, we are unable to evaluate the performance of our breast tissue-specific model in other tissues such as blood. Furthermore, our epigenetic analysis was performed on whole breast issue and did not account for specific cell types in the normal breast. Future studies are needed to assess whether and how DNAm age differs among different cell types of the normal breast.

## Conclusions

In this study, we developed a breast tissue-specific model to estimate DNAm age using next-generation sequencing data. Breast tissue-specific DNAm age was calculated based on the methylation of a novel set of 286 clock CpGs that were selected by a penalized regression model trained on data from normal breast tissue. We found that the estimated DNAm age was highly correlated with chronological age with a minimal error. We used this model to explore epigenetic aging in different breast tissue types, including normal, adjacent-normal, and tumor breast tissue. We observed that epigenetic age acceleration was significantly higher in breast tumor tissue than that of normal or adjacent-normal tissue, while there was no significant difference between normal and adjacent-normal breast tissue. We have also observed that epigenetic age acceleration in breast tumors appeared to be associated with distinct tumor clinical features including molecular subtype, grade, and stage. Further, the decreased epigenetic age acceleration in aggressive molecular subtypes and more advanced diseases is of note. While larger studies are needed to confirm these findings, future research could explore the following questions: whether our breast tissue-specific model of DNAm age estimation can be applied to the blood and other easily accessible tissues, such as saliva, buccal cells, and serve as a surrogate marker of breast aging; whether and how breast tissue-specific DNAm age is influenced by various factors, especially by known breast cancer risk factors; and if breast tissue-specific DNAm age could be used as a predictive biomarker for cancer development, treatment response, and survival.

## Methods

### Breast tissue samples

The main aim of the study is to develop a model to estimate breast tissue-specific DNAm age. We developed the model using data from normal breast tissue. Our study included 459 normal breast tissue samples from healthy women who were randomly selected from a pool of women who donated both blood and normal breast tissue samples to the Komen Tissue Bank between 2005 and 2009 and were free of breast cancer up to the time of donation. These participants encompass a wide range of ages (18–83 years), reproductive history, and lifestyle exposures. See Additional file 2 (Supplementary Table 1). Such diversity ensures the robustness of our model to estimate breast tissue-specific DNAm age. In order to apply our developed model to estimate DNAm age in other breast tissue types, separately, we included 107 primary breast tumor and 45 matched histologically normal breast tissue (adjacent to primary tumor site) samples from breast cancer patients with untreated tumors from the Indiana University Simon Cancer Center (IUSCC) Tissue Bank. These cases are patients with pathologically confirmed primary breast cancer diagnosed at one of three hospitals in Indianapolis, IN, between 1998 and 2009: University Hospital, Wishard Hospital, and IUSCC. All breast tissue samples were snap-frozen in liquid nitrogen within 5 min of removal and determined to be of high quality through histological and molecular quality control tests. Tumor samples were pathologically verified for high tumor content. Table 1 presents age distribution of breast tissue dataset in the study. While the mean chronological age was similar in the training and the testing data sets of normal breast tissue (*p* = 0.99), breast tumor and adjacent-normal tissue samples were approximately 5-7 years older than normal breast tissue samples on average (*p* = 7.5 × 10^−5^ and 0.02, respectively). 

### DNA extraction and breast tissue-specific DNA methylation profiling

Genomic DNA was extracted from freshly frozen normal, tumor, and adjacent-normal breast tissue samples using the Qiagen DNeasy Blood and Tissue Kit (Qiagen Inc., Venlo, Netherlands). Extracted DNA was first evaluated for its quantity and quality using Agilent TapeStation 4200 (Agilent Technologies, Santa Clara, CA, USA) electrophoresis and Thermo Fisher Qubit 3.0 (Thermo Fisher Scientific, Waltham, MA, USA) flurometry technologies. Genome-wide DNAm profiling was performed using the Illumina TruSeq Methyl Capture EPIC Library Prep Kit [41] and next-generation sequencing technology for genomic DNA sequencing. Five hundred nanograms of high-quality genomic DNA were used for library preparation. Specifically, DNA library preparation first included fragmentation to an average size of 150–200 bp using a Covaris S2 ultrasonicator (Covaris Inc., Wobnurn, MA, USA), followed by end-repair, 3’ A-tailing, and adaptor ligation. Libraries were then pooled in groups of four in equal aliquot, on which two rounds of hybridization and capture using Illumina-optimized EPIC probe sets (covering > 3.3 million targeted CpG sites), bisulfite conversion, and amplification were performed. Five percent PhiX DNA (Illumina Inc.) was added to each library pool during cluster amplification to boost diversity. Construction of DNA libraries and subsequent processing and DNA sequencing of paired-end reads (2 × 100 nt reads) were performed according to the standard Illumina protocol using the HiSeq4000 sequencing systems.

### DNA methylation sequencing data pre-processing

Raw sequencing reads were trimmed to remove both poor-quality calls and adapters using Trim Galore! v0.4.4 [42]. Trimmed reads were then aligned to the Genome Reference Consortium human genome build 37 [43] using Bismark v0.19.0 [44]. Duplicated reads were removed and cytosine methylation calls were extracted from the deduplicated reads. Methylation calls that overlap with the Illumina EPIC-seq targets were used in downstream analyses. Deduplicated reads on each cytosine locus were used to determine the DNAm levels (*β* values); a *β* value is evaluated as the ratio of the number of sequenced methylated cytosine reads to the total number of reads for each locus. Thus, *β* values range from 0 (completely un-methylated) to 1 (completely methylated). To ensure high quality data, samples with > 20% missing CpGs were excluded, including three normal and two tumor breast tissue samples. A CpG was included if it had a *β* value determined with ≥ 10 total reads, had < 10% missingness across samples, and was present in each tissue-type data set. After these QC steps, a total of 2,471,574 CpGs remained for downstream analyses.

Imputation was performed after QC to recover any residual missing CpG *β* values. Since cancer has been shown to affect DNAm patterns [45, 46], missing CpG *β* values were imputed separately for each tissue type data set. Missing CpG probe *β* values were recovered using *k*-nearest neighbor (kNN) imputation as implemented in the R-Bioconductor package “impute” [47]. Since studies have shown that the methylation levels of neighboring CpG sites are more likely to be co-methylated [48–51], methylation matrices were sorted by chromosome and base-pair position prior to kNN imputation to maximize the likelihood of the algorithm selecting the optimum neighbors for imputation.

### DNAm age estimation using a penalized regression model

Since cancer is known to have profound effect on DNAm levels, we used only normal breast tissue from healthy women to construct the model for DNAm age estimation. Specifically, normal breast tissue samples were randomly divided into training (*N* = 368) and testing (*N* = 91) data sets. Chronological ages were transformed by the function below prior to model training [6]:

$$ f(x)=\left\{\begin{array}{c}\log \left(x+1\right)-\log \left(C+1\right),x\le C\\ {}\frac{x-C}{C+1},\kern8.5em x>C\end{array}\right.. $$

Here, *C* is set at 20 years for humans. This transformation was performed to provide additional stability during model training. The training data set was then used to regress the transformed chronological ages on the methylation levels of approximately 2.4 million profiled CpGs using a penalized regression model implemented in the R package “glmnet” [52]. The alpha parameter for the model was chosen as 0.5, for the elastic net algorithm, and the lambda parameter was determined by the average of 100 iterations of 10-fold cross validation (lambda = 0.0343). After successful training of the model, values that the model predicted for each sample were converted into DNAm ages using the inverse of the above age transformation function (Additional file 3). We validated the DNAm age estimator defined in the training dataset in the independent testing data set of normal breast tissue.

To assess the accuracy of DNAm age estimates from our model, we considered two measures: the Pearson correlation coefficient between the estimated DNAm age and chronological age and the absolute median predictive “error,” defined as the median absolute difference between the estimated DNAm age and the chronological age.

We further examined age-adjusted DNAm levels of the 286 clock CpGs across all samples from the three breast tissue types. We obtained DNAm residuals for each CpG locus by regressing *β* values on chronological age and visualized how DNAm levels changed across samples and across tissue types. Analyses were also performed to identify clock CpGs that were differentially methylated across tissue types. A clock CpG was considered significantly and differentially methylated between two tissue types if it had a mean |∆β| > 0.1 with *p* < 0.05.

### DNAm age estimation and epigenetic age acceleration in different breast tissue types

We applied our model to estimate DNAm age in different types of breast tissue, including normal, tumor, and adjacent-normal breast tissue. We further investigated three measures of epigenetic age acceleration: epigenetic age acceleration rate defined as the increase in DNAm age per unit increase in chronological age, epigenetic age acceleration difference defined as the difference between estimated DNAm age and chronological age, and epigenetic age acceleration residual defined as the residual of a linear model that regresses DNAm age on chronological age. For a given tissue type, a Wilcoxon rank-sum test was used to determine whether DNAm age was significantly higher or lower than chronological age, or EAAD value was significantly different from zero.

Because aging significantly influences changes in DNA methylation, DNAm age is highly correlated with chronological age [6, 21, 53]. Thus, when comparing DNAm age across different groups, confounding by chronological age difference between the groups needs to be considered and accounted for. While the mean chronological age was similar in the training and the testing data sets for normal breast tissue (*p* = 0.99), breast tumor and adjacent-normal tissue samples were approximately 10 years older than normal breast tissue samples on average (*p* = 7.5 × 10^−5^ and 0.02, respectively). We utilized two approaches to account for the confounding effects by chronological age when comparing epigenetic age acceleration across tissue types in our study. First, we performed age-matched paired analyses and examined the difference of the estimated DNAm ages between two groups. A Wilcoxon rank-sum test was used to determine if the DNAm age difference between the two groups was significantly different from zero. For the comparison between tumor and adjacent normal breast tissue, since the samples are paired from the same woman, a Wilcoxon signed rank test was used instead. This approach controls of confounding by age but may result in a smaller sample size and reduced study power due to unmatched samples. Second, we performed analyses using EAAR values that inherently control for chronological age. The interpretation of EAAR values within a single group, however, is less straightforward when compared to the interpretation of EAAD values and, as such, are only considered when assessing relative differences between groups. A Kruskal–Wallis one-way ANOVA test followed by a post hoc Dunn’s test was used to test for significant differences in the location parameters for the EAAR distributions between groups. This approach maximizes sample size and study power when compared to the age-matched paired analysis.

### DNAm age estimation in breast tumor tissue with distinct clinical characteristics

DNAm age was also estimated in breast tumor subgroups with distinct clinical characteristics, including molecular subtype, Scarff-Bloom-Richardson [54] tumor grade, and tumor stage. For each tumor subgroup, we calculated EAAD values based on the estimated DNAm ages, and tested if the median EAAD value was significantly different from zero using a Wilcoxon rank-sum test. Given the smaller sample size in tumor subgroups, we used EAAR values to facilitate the comparisons across tumor subgroups to maximize the study power and control for confounding by chronological age. A Kruskal–Wallis one-way ANOVA test followed by a post hoc Dunn’s test was used to test for significant differences in the location parameters for the EAAR distributions across tumor subgroups.

All *p* values were based on two-sided tests and were considered statistically significant if *p* < 0.05. Statistical analyses were performed using the R software version 3.5.1 (https://cran.r-project.org).

## Supplementary information

**Additional file 1.** Coefficient values for the breast tissue-specific DNAm age model. This comma-delimited value (csv) text file contains the genomic location, model coefficients, and gene annotations for the set of 286 clock CpGs derived in this study.

**Additional file 2.** Supplementary Table 1. Characteristics of healthy women participants who contributed normal breast tissue samples to the current study.

**Additional file 3.** R software tutorial. This file contains R software (https://cran.r-project.org/) which reads in Additional file 1 as well as user-provided DNAm data and returns the estimated DNAm ages for each sample within the user-provided data set.

## Data Availability

The datasets used and/or analyzed during the current study are available from the corresponding author on reasonable request.

## Abbreviations

ATM: Ataxia-telangiectasia mutated
CpG: Cytosine-phosphate-guanine
DNAm: DNA methylation
EAAD: Epigenetic age acceleration difference
EAAR: Epigenetic age acceleration residual
EGF: Epidermal growth factor
EPIC-seq: Illumina TruSeq Methyl-Capture EPIC Library Prep Kit and next-generation sequencing
ER: Estrogen receptor
HR: Hormone receptor
IPA: Ingenuity pathway analysis
IUSCC: Indiana University Simon Cancer Center
K: Komen normal breast tissue
kNN: *k*-nearest neighbor
N: Histologically normal breast tissue (adjacent to primary tumor site)
NAD: Nicotinamide adenine dinucleotide
QC: Quality control
T: Primary breast tumor tissue
TNBC: Triple-negative breast cancer
