# Supplementary material for: Estimating breast tissue-specific DNA methylation age using next-generation sequencing data
Source: Clin Epigenetics. 2020 Mar 12;12:45. doi: 10.1186/s13148-020-00834-4 (PMC7282053; doi:10.1186/s13148-020-00834-4)
Supplement: Supplementary file 2 — Additional file 2. Supplementary Table 1. Characteristics of healthy women participants who contributed normal breast tissue samples to the current study. [file 13148_2020_834_MOESM2_ESM.pdf]

Supplementary Table 1. Characteristics of healthy women participants who contributed normal breast tissue samples to the current study. Distribution: mean ± standard deviation and percentage for continuous and categorical variables, respectively.

**Characteristic**	**Distribution**
Age at Sample Collection	47.7 ± 13.4 years
Race	African American (61%)/Caucasian (39%)
Body Mass Index (BMI)	31.8 ± 8.2
Age at Menarche	12.5 ± 1.7 years; <11 (12%), 11 (12%), 12 (27%), 13 (24%), >14+ (24%) years
Age at First Birth	24.4 ± 5.9 years; <20 (23%); 20-24 (34%), 25-29 (23%), 30-34 (13%), 35+ (7%) years
Parity	Nulliparous (29%)/Parous (71%); 1-2 (48%), 3-4 (20%), 5+ (3%)
Menopause Status	Pre-menopausal (49%)/Post-menopausal (51%)
Age at Natural Menopause	44.0 ± 7.8 years
Alcohol Use	Yes (55%)/No (45%)
Tobacco Use	Yes (9%)/No (91%)
Multivitamin Use	Yes (47%)/No (53%)
Location	Urban (89%)/Rural (11%)
